# Letter from New York

**Published:** 1856-12

**Authors:** P. Pineo


Communicated for the Boston Medical and Surgical Journal.

*Letter from New York.* By P. PINEO, M. D.

I listened to a clinic on *gangrene of the lungs* and *cirrhosis of the liver*, by Prof. Clarke, of the College of Physicians and Surgeons. He is a fluent and pleasant lecturer, and evidently a good teacher. He said that gangrene of the lungs was never the result of pnuemonia, although pneumonia was often the consequence of gangrene. In the case presented there was the somewhat unusal occurrence of gangrene in both lungs. In one lung pneumonia was present while it was absent in the other.

The true cause of gangrene of the lungs he thought was always obstruction or obliteration, either from calcareous deposit or something else, of the artery supplying the part; and the same was true of gangrene of of other portions of the body. The number of cases that get well is about fifty per cent. There had been two cases in the hospital this season, one of which recovered; the other furnished the pathological specimen which was shown to the students.

The symptoms diagnostic of the disease are, the intolerable fœtor, expectoration of the peculiar dirty-green matter, and rapid prostration. The treatment to be relied upon is a supporting one—tonics, and as much nourishing food as you can stuff into the patient, or his digestive organs will bear.

*Cirrhosis of the liver*, or atrophy and induration, is the result of ininflammation of the capsule of Glisson. This may arise from any cause which will occasion inflammation in any other part—but the habitual use of ardent spirits is the most frequenting exciting cause; hence the name of *gin drinker's liver.*

Prof. Van Buren, of the University Medical College, delivered a clinic on Gonorrhœa. He considers copaiva almost a specific for the disease when judiciously administered. It should be given in very small doses at first, and gradually increased as the stomach will bear it. In order to cure the disease, Dr. Van Buren says it is indispensable that the medicine should be continued for at least ten days after the discharge has ceased. Cubebs, though greatly inferior, may be resorted to when the stomach will not tolerate copaiva. Injections he would use, though very mild ones. A weak solution of plumbi subacetas he thought better than any other. He warned the class against giving strong injections of the more stimulating articles, such as argenti nitras, &c. He thought there was great danger of evil ensuing in the form of stricture, &c.

Additions are being made to Bellevue Hospital, which when completed will contain fourteen hundred beds. Through the politeness of Dr. Gouley, a very promising young anatomist, who filled the chair of anatomy at the Woodstock Medical College last spring, I was shown the new dead house, the culinary arrangements of the hospital, &c. Thousands of tons of coal are placed in the grounds of the Hospital for gratuitous distribution to the poor. The institution is a great charity and reflects much credit on the city of New York. There is a ward in the Hospital devoted almost altogether to ulcers on the extremities. A large number of cases were treated by opium, as recommended by Mr. Skey, surgeon to St. Bartholomew's Hospital. Cold water dressings were applied to the part, and a pill of soap and opium given three times a day, the patient getting from one-half to a grain of opium each dose. A good nourishing diet was allowed. The improvement in every case was positive and rapid. A few cases were treated with the actual cautery, and with good results.

In the syphilitic ward at the City Hospital can be seen all the forms of syphilis, from the simple chancre to the most revolting constitutional affections of the bones. The surgeon removed, from the forehead of a patient, a portion of the outer table of the skull, measuring three inches in length and two in breadth. The inner table was perforated by caries, and a probe was passed into the brain. Surely, any young man who visits the syphilitic wards of a New York hospital, will be deterred from exposing himself in any way by which he may possibly contract so loathsome, vile and wretched a disease.

*New York, October,* 1856.

